# May the odds be ever in your favour

**DOI:** 10.1111/test.12162

**Published:** 2018-05-09

**Authors:** Laura J. Bonnett, Simon R. White

**Affiliations:** Department of Biostatistics, University of Liverpool, Liverpool, UK; MRC Biostatistics Unit, University of Cambridge, Cambridge, UK

**Keywords:** Teaching statistics, practical activity, two-dice experiment, probabilities, simulations

## Abstract

Probability and chance are essential concepts, not just in statistics but in real life. We present an adaptable activity which investigates what we mean by bias, how we can identify bias, and how we can use it to our advantage!

## Introduction

In the authors’ experience, ask a secondary school student in the United Kingdom (age 11 to 18) what they understand by probabilities and chance and it is likely they will respond that probability relates to counters in a bag or socks in a drawer. We are keen to present the concepts in a fresh and inspiring way.

As working statisticians, our engagement with the next generation of possible statisticians usually takes place at science fairs such as The Big Bang and Cambridge Science Festival. These events require quick-hitting low-technology activities which immediately entice students to the stall, engage them and can then introduce and demonstrate a statistical concept in less than 5 min. Therefore, we have to move away from the student-perceived ‘dull’ counters in bags and socks in a drawer demonstrations whilst still teaching the concept of probability and chance.

Games are often thought of as a distraction – something to be done for fun. However, there is growing evidence to suggest that games can do much more, especially when it comes to classroom learning. A review of research into the effectiveness of games for educational purposes determined that mathematics was the subject area with the greatest percentage of results favouring games over conventional classroom instruction (Randel et al. 1992).

An example of an existing activity developed for the purpose of statistical outreach to illustrate probability involved the Hunger Games, an annual event in the fictional country of Panem (Caudle and Daniels 2015). The authors used statistical analysis and computer simulations to explore the possibility that the Gamemakers, those in charge of planning the Hunger Games, fixed the lottery. Additionally, a recent informal study introduced undergraduate students to two versions of a story involving probability (Passaretti and Vistocco 2017). The results of this study revealed the students had trouble detecting equal probabilities in a sampling scheme without replacement in which no information on earlier draws was available.

We therefore propose a novel activity to demonstrate chance via biased die and an outdoor-sized Ludo board game. The learning aim is to understand the concept of bias and determine when the method of estimation of probabilities is biased, and why that can be useful in real life applications. Our activity can be tailored to the available time, context and level of the target audience. In the following sections, we present a suggested template for delivering the activity as a single game play, and as a multiple game play version, together with additional extension activities.

## Materials

**Fig. 1:**
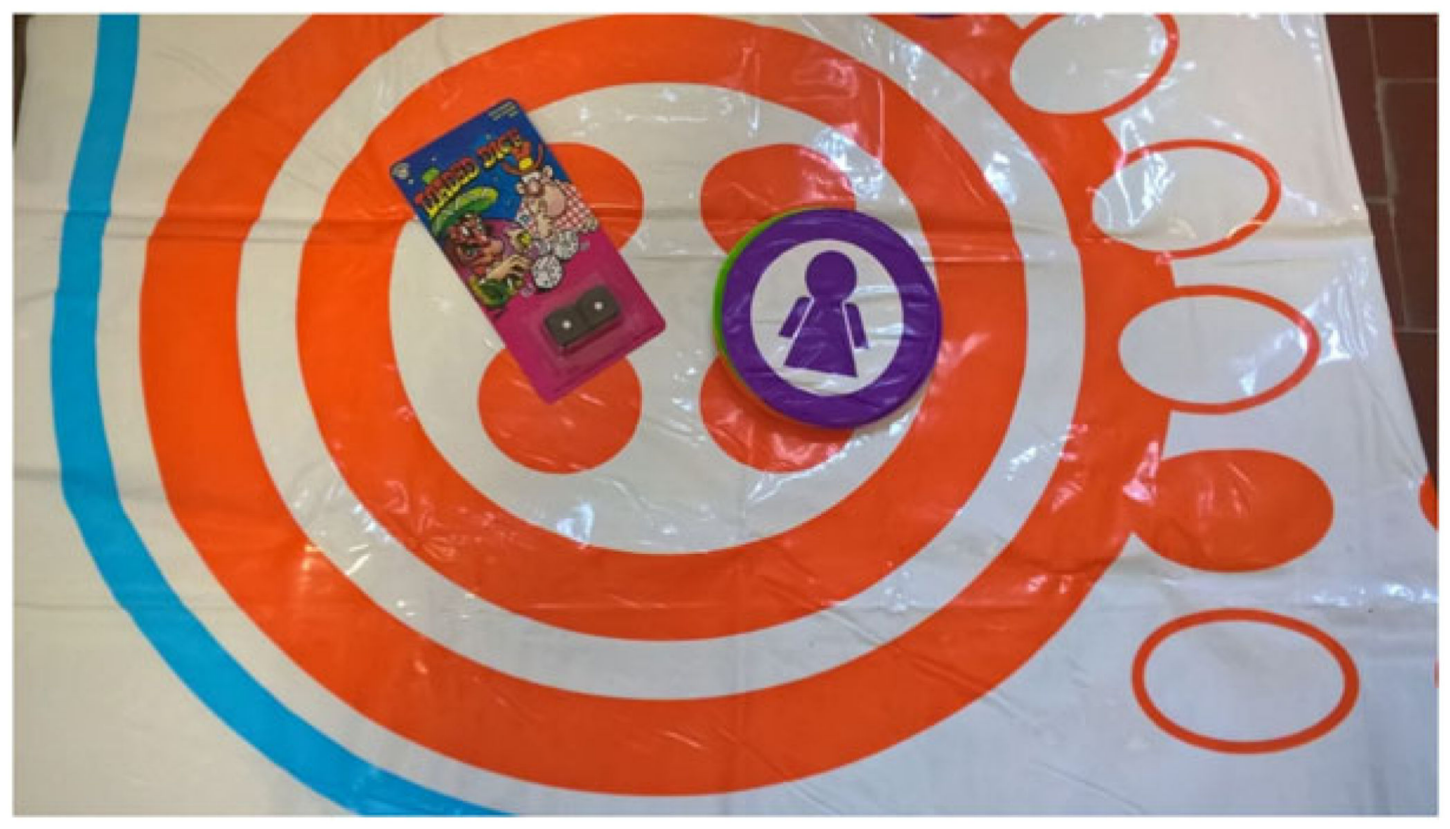
Example of required materials - a Ludo board, counters and two identical-looking dice of which one is fair and one is biased. [Colour figure can be viewed at wileyonlinelibrary.com]

Our activity requires as large a Ludo board as possible – the outdoor versions are best – with counters, at least one biased die (available from joke shops and online retailers) and one identical-looking fair die (Figure 1). These resources are fairly easy to source ensuring that the activity is ideal for science festivals. The classroom-based version of this activity requires only one biased and one fair die per subgroup of students.

## Single Game Play

The first element of this activity requires a discussion regarding bias. Have the students heard of the word biased before? If so, what do they understand by that term? If not, we suggest turning the question on its head and asking what random means, specifically in terms of a fair die. The answers we help students to give are that a fair die is one which has a (fairly) equal chance of landing on any number from 1 to 6, whilst a biased die is one which lands on one number more often than would be expected by chance.

**Fig. 2:**
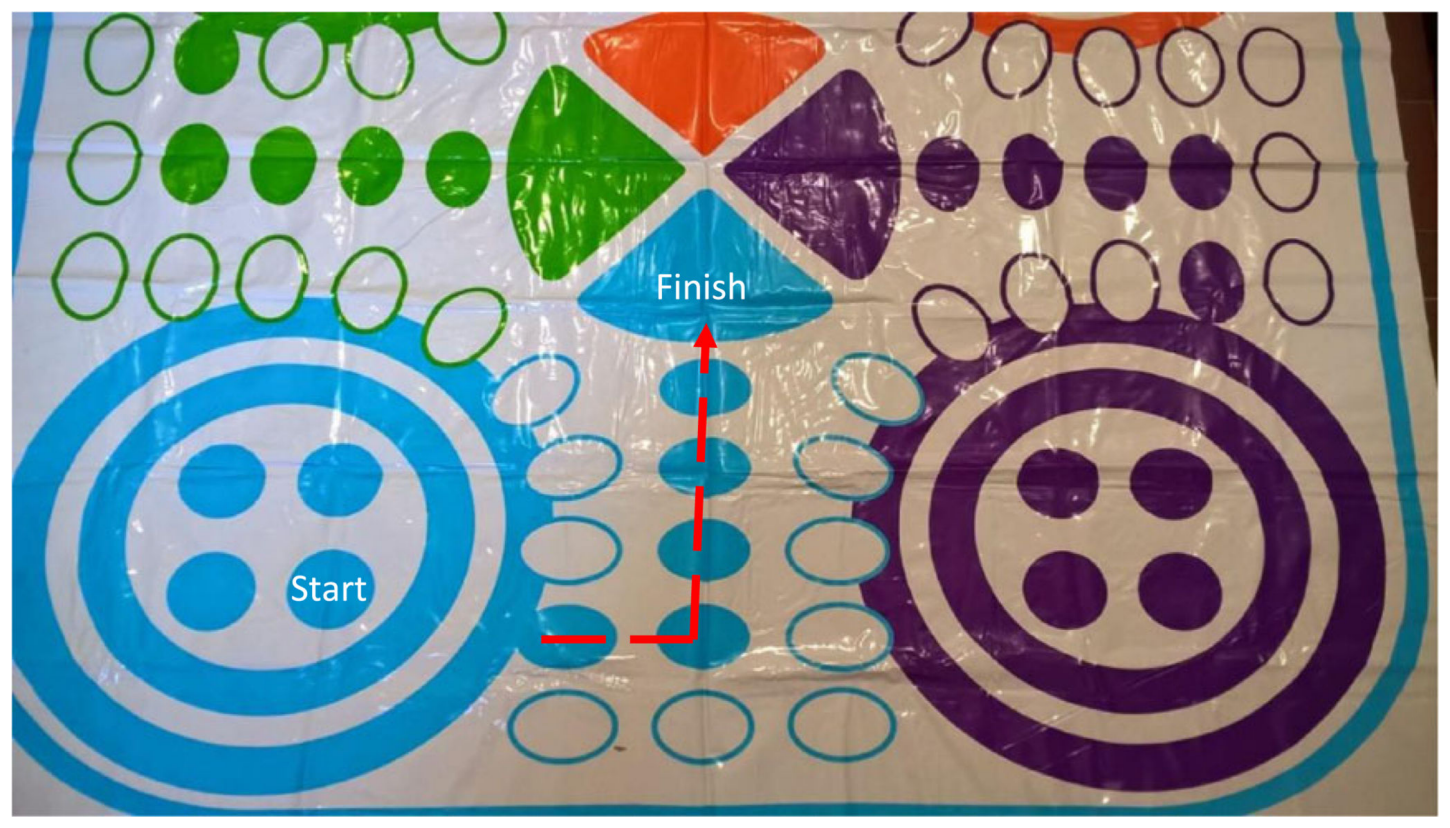
Ludo board with the suggested shortened track, start and finish points marked. [Colour figure can be viewed at wileyonlinelibrary.com]

Next, we explain the rules of Ludo as follows. Participants must roll a 6 to start the game. If a 6 is not rolled, the turn passes to the next player. Once a player has rolled a 6, the participant moves their token forward along the track the number of places indicted by the die roll. If a participant rolls a 6, they get a bonus roll. If the additional roll results in a 6 again, they get an additional bonus roll. The first player to bring their token to the finish wins the game. Figure 2 demonstrates the shortened track we use at science fairs to ensure that the activity is of an appropriate duration for the setting.

Having explained how the game works, the participants should now be given the two dice (one fair and one biased) and asked to select which die they wish to use to play the board game Ludo. Students are encouraged to roll both dice multiple times to determine if either die leads to a particular outcome more frequently than expected by chance. Participants should also be stimulated to think whether the die is biased in their favour or against them – they might need reminding that if the die lands on a 6 more often than the numbers 1–5, then it is advantageous for Ludo where the participant needs a 6 to begin the game and receives bonus rolls as a reward for rolling a 6. Once the participants have chosen their preferred die, another student in the group, or the activity coordinator, should be given the other die to use for the game.

The students then play the game using their selected dice, with reminders and encouragement given as required. At the end of the game, the biased die is revealed, and a discussion is encouraged identifying the direction of the bias – what number appeared most often when this die was rolled during the game?

Assuming that the die is biased towards a 6, there are four outcomes from a single game play – the students may have correctly selected the biased die and used it to win their game of Ludo. However, they may have correctly selected the biased die and still lost at their Ludo game, or they may have selected the fair die instead of the biased die and either won or lost at Ludo. The reasoning behind the outcome in their particular game should be explained to conclude the activity. This can also be illustrated through a classroom version of the game which includes the following simulation study.

## Simulation Game Play

In the single game play version, we encouraged students to roll both dice multiple times to estimate if one led to a particular outcome more often than expected. This introduced them to the concept of a simulation study – a way of considering what happens over repeated games. In the classroom version of this activity, we encourage students to undertake a simple simulation study considering the outcome of both the biased die and the outcome of the fair die over 50 rolls of each. A fair die has an equal probability (p=16) of landing on each number. This is not true of a biased die which has an unequal chance of landing on each number. Assuming that the die is biased towards a 6, the probability of a 6 (or any number) will be different for each biased die. Additionally, not all biased dice are biased to a 6. Results based on our dice which is biased to a 6 can be seen in Table 1.

Rolling the die multiple times, such as in a simulation study, helps participants to determine which die lands on the same number more often than it should. Rolling the die multiple times also enables the participant to estimate the probability of getting each number – keep a tally of the numbers rolled for 30, 50, 100 … rolls, as shown in Table 1. The number of rolls of each number divided by the total number of rolls will give the estimated probability of getting that number. For example, if the student rolled the die 50 times as above and rolled a ‘1’ five times, then the probability of a ‘1’ using that die is 550=110=10%.

Students may be surprised to see that the fair die did not lead to almost equal probabilities of each outcome. Intuitively, we might expect that five rolls of a fair die should come up with a different outcome each roll. However, we can prove that the chance of all five rolls being different is in fact small, as follows.

On the first roll, the probability of a different number is clearly 66=1 as no outcome will result in a duplicate given that this is the first roll. For the second roll, the chance of the outcome being different from the first is 56 as there is now one less value that we have not seen before. For example, if we rolled a 6 on the first roll, then a different outcome for roll two compared with roll one can only be the values 1 to 5. The third roll has a 46 chance of leading to a different outcome based on similar logic and so on. Hence, the total probability of all five rolls turning out different is

66×56×46×36×26=1×56×23×12×13=554≅9.3%

by probability of intersections using conditional probability (Mendenhall et al. 2012). Therefore, if we roll a die five times in a row, we would expect over 90% of the rolls to contain duplicates.

The results for the biased die are also potentially surprising. Clearly, our die favours a 6, shown by the high percentage for this outcome in Table 1. It also rolls a 1 less frequently than expected by chance due to 1 being in the opposite location to a 6 on a die and the location for the weight within the die. However, the outcomes 2 to 5 have fairly equal probabilities of being the outcome – students usually expect the biased die to land on a 6 every roll. However, this is highly unlikely (see fair die explanation).

An extension of the simulation study is to consider other variations such as angle of throw, speed of throw, surface die rolls on and so forth as these all also have the potential to influence the results.

A good way to conclude the activity is by discussing the real-life application of the task the students have undertaken. As medical statisticians, our application of choice is usually related to our area of interest. However, this can clearly be tailored to the presenter, or other topics in the curriculum as required. Our preferred application is as follows. Unequal probabilities of selection, as obtained from a biased die for example, can be used in clinical trials where the aim is to recruit people to treatment groups of different sizes – biasing the probabilities to the relevant proportions helps to ensure different sized groups of participants. An additional application would be stratified sampling to ensure that people are selected at random but proportional to the county or city they live in for example – biased probabilities could be used to ensure over-sampling of relevant areas.

## Conclusion

Probability and chance are essential concepts both within statistics and within real life. Even a basic understanding of probability enables us to comprehend weather reports, sports results, the chance of being struck by lightning, winning the lottery and so on. Our experience suggests that students associate probability with so-called dull experiments involving counters in a bag and socks in a drawer. We therefore propose a novel way of considering probability which can be utilized as a quick-hitting activity at a science fair or as a more in-depth classroom activity.

Both incarnations of the activity introduce students to the concept of randomness and bias via dice and the traditional (large-scale) board game of Ludo. Students are encouraged to identify the biased die by rolling it a few times or more thoroughly through a simulation study. In particular, they are required to think about whether the biased die is biased in their favour for this particular game or against them. By convincing students that dice can be biased in their favour and the real life benefits of this bias, we hope to get them thinking about probabilities in a more positive and engaging way.

## Figures and Tables

**Table 1:** Results from 50 rolls of a fair and a biased die

			Outcome – *n* (%)			
Die	1	2	3	4	5	6
Fair	5 (10)	7 (14)	6 (12)	13 (26)	7 (14)	12 (24)
Biased	2 (4)	5 (10)	7 (14)	6 (12)	8 (16)	22 (44)
